# Pyroptosis-related genes regulate proliferation and invasion of pancreatic cancer and serve as the prognostic signature for modeling patient survival

**DOI:** 10.1007/s12672-022-00495-0

**Published:** 2022-05-28

**Authors:** Wenjing Song, Zhicheng Liu, Kunlei Wang, Kai Tan, Anbang Zhao, Xinyin Li, Yufeng Yuan, Zhiyong Yang

**Affiliations:** 1 Department of Hepatobiliary and Pancreatic Surgery, Zhongnan Hospital of Wuhan University, Wuhan, China; 2 Pancreatic Surgery Center, Zhongnan Hospital of Wuhan University, Wuhan, China; 3 Clinical Medicine Research Center for Minimally Invasive Procedure of Hepatobiliary & Pancreatic Diseases of Hubei Province, Wuhan, Hubei China

**Keywords:** Pyroptosis, CASP4, NLRP1, Pancreatic ductal adenocarcinoma, Gene signature, Prognosis

## Abstract

**Objective:**

Pancreatic ductal adenocarcinoma (PDAC) has high mortality and poor prognosis. Pyroptosis can influence the prognosis of patients by regulating the proliferation, invasion, and metastasis of cancer cells. However, the role of pyroptosis-related genes (PRGs) in PDAC remains unclear.

**Methods:**

In this study, based on the Cancer Genome Atlas (TCGA) cohort of PDAC samples, univariate Cox analysis and LASSO regression analysis were used to screen the prognostic PRGs and establish the gene signature. To further evaluate the functional significance of CASP4 and NLRP1 in PDAC, we also conducted an in vitro study to explore the mechanism of CASP4 and NLRP1 regulating the occurrence and development of PDAC. Finally, we investigated the relationship between CASP4 and NLRP1 expression levels and drug sensitivity in pancreatic cancer cells.

**Results:**

A risk prediction model based on CASP4 and NLRP1 was established, which can distinguish high-risk patients from low-risk patients (P < 0.001). Both internal validation and external GEO data sets validation demonstrate good predictive capability of the model (AUC = 0.732, AUC = 0.802, AUC = 0.632, P < 0.05). In vitro, CCK8 and Transwell assay suggested that CASP4 may accelerate the progression of PDAC by promoting proliferation and migration of pancreatic cancer cells, while NLRP1 has been found to have tumor suppressive effect. It should be noted that knockdown of CASP4 reduced the level of coke death, the expression levels of acetyl-CoA carboxylase, FASN, SREBP-1 and SREBP-2 were decreased, and the number of lipid droplets was also significantly reduced. Moreover, the enrichment of signaling pathways showed that NLRP1 was significantly correlated with MAPK and RAS/ERK signaling pathways, and knocking down NLRP1 could indeed up-regulate p-ERK expression. Finally, high expression of CASP4 and low expression of NLRP1 increased the sensitivity of pancreatic cancer cells to ERK inhibitors.

**Conclusions:**

In especial, CASP4 can promote tumor progression by promoting the synthesis and accumulation of fatty acids, while NLRP1 acts on RAS/ERK signaling pathway. Both of genes play an important role in the diagnosis and treatment of PDAC, which may also affect the inhibitors of MAPK/ERK efficiency.

**Supplementary Information:**

The online version contains supplementary material available at 10.1007/s12672-022-00495-0.

## Introduction

Pancreatic ductal adenocarcinoma (PDAC) is an exceptionally lethal malignancy and 5-year relative survival rate is 11%. It is estimated that There will be 62,210 (3.24%) new cases of pancreatic cancer and 49,830 (8.18%) deaths in the United States in 2022. According to statistics, pancreatic cancer was the fourth leading cause of cancer death in the United States in 2019 [1]. By 2025, pancreatic cancer is estimated to become the third leading cause of cancer death in European countries and the second most deadly cancer in the United States [2, 3]. The only cure for PDAC is early detection followed by surgical removal. However, because of the insidious onset, difficulty in early diagnosis and rapid progress of PDAC, most patients have lost best opportunity to operate at the time of diagnosis. Although FOLFIRINOX, modified FOLFIRINOX and nab-paclitaxel plus gemcitabine regimens have demonstrated better response rates, developing more effective treatments remains challenging [4]. Advances in high-throughput sequencing technology and systems biology are contributing to a better understanding of the underlying molecular mechanisms of PDAC and the search for new molecular targets and corresponding therapies to prolong the survival time of patients [5, 6]. In addition, different PDAC subtype classification systems can be established according to the gene characteristics of the tumor at the molecular level, so as to predict the prognosis of patients and select therapeutic drugs [7–10].

Pyroptosis, a kind of programmed cell necrosis that has attracted much attention recently, performed through Gasdermin (GSDM) protein family directly [11]. Activated caspase releases the structural domain with the activity of binding membrane phospholipid membrane drilling through cleaving GSDM protein, thus inducing pyroptosis [12]. Different from apoptosis which is characterized by immune silencing, pyroptosis shows rupture of cell membrane and the release of many cytokines and danger signaling molecules, which activate the immune system and lead to inflammatory response [13, 14]. Pyroptosis is closely related to various diseases, especially malignant tumors [15], where pyroptosis may play a dual role. On the one hand, pyroptosis can activate the anti-cancer immune response and inhibit the occurrence and progression of cancer. On the other hand, pyroptosis, as a means of pro-inflammatory death, can promote the formation of the tumor microenvironment (TME) suitable for tumor cell growth and accelerate cancer growth [16]. Recently, characteristics of pyroptosis-related genes (PRGs) have been shown to be significantly associated with prognosis in ovarian cancer, lung adenocarcinoma, and gastric cancer [17–19]. Therefore, in recent years, researchers have attempted to combine PRGs with various tumor treatments to eliminate malignant cells by regulating pyroptosis [20]. Specifically, A large number of reports have shown that chemotherapy drugs and miRNA can induce the pyroptosis of tumor cells, thus inhibiting the malignant progression of tumor [21–24]. Therefore, it is of great significance to study the relationship between PRGs and prognosis and to explore its expression characteristics and functional involvement in PDAC.

Based on the RNA-seq data and clinicopathological characteristics of TCGA-PAAD dataset, we proposed the signature of PRGs and validated the model by using multiple groups of patient data. In addition, we described the expression of the gene signature at the protein level, and deeply studied the signaling pathway and biochemical process involved in the PRGs. Besides, we inferred and studied the other functions of PRGs in pancreatic cancer cells in vitro further. Moreover, we analyzed the relationship between PRGs and the drug sensitivity in PDAC, which we hope can contribute to prognostic monitoring and treatment strategies for PDAC patients.

## Materials and methods

### Patient data acquisition

We downloaded the RNA-seq, gene mutation data of the pancreatic cancer sample (TCGA-PAAD) and relevant clinical data of the patient from the TCGA database (https://portal.gdc.cancer.gov/) on July 10, 2021. RNA-seq data and clinical information from the external validation cohort (ID: GSE62452, GSE57495) were obtained from the Gene Expression Omnibus (GEO) database (https://www.ncbi.nlm.nih.gov/geo/) [25, 26]. After data cleaning, 173 patients in TCGA-PAAD, 64 patients in GSE62452, and 63 patients in GSE57495 had complete survival data. The clinicopathological characteristics of patients in different cohorts were shown in Table 1, including survival time, survival status, grade, stage, and TNM. 7 paired confirmed PDAC tissues and adjacent normal tissues were obtained from patients who underwent pancreaticoduodenectomy in Zhongnan Hospital of Wuhan University with informed consent. None of the patients had received radiotherapy, chemotherapy or immunotherapy before surgery and this study was approved by the ethics committee of Zhongnan Hospital.

**Table 1:** The clinicopathological characteristics of patients

Characteristics	TCGA-PAAD (n = 173)	GSE62452 (n = 64)	GSE57495 (n = 63)
Status			
Alive	115	15	21
Dead	58	49	42
Grade			
Grade 1	30	2	
Grade 2	92	32	
Grade 3	47	29	
Grade 4	2	1	
Unknown	2	0	
Stage			
Stage I	20	4	13
Stage II	143	44	50
Stage III	3	10	0
Stage IV	4	6	0
Unknown	3	0	0
T			
T1	7		
T2	23		
T3	138		
T4	3		
Unknown	2		
N			
N0	48		
N1	120		
Unknown	5		
M			
M0	79		
M1	4		
Unknown	90		

### Gene signature identification and score construction

Based on previous studies and reviews, we extracted 33 PRGs [16, 17, 27–29], as shown in Table 2. In order to evaluate the prognostic value of PRGs, univariate Cox regression analysis was used to evaluate the correlation between each gene in the TCGA-PAAD cohort and the survival time and survival status of patients. Finally, 11 prognostic PRGs were identified for further analysis. Elastic net regularization and least absolute shrinkage and selection operator (LASSO) regression analysis was used to identify independent prognostic genes strongly associated with overall survival (OS) in PDAC patients and calculate risk scores. The risk score was calculated by the following formula:

$$\text{Risk} \; \text{score}={\sum }_{i=1}^{n}Coef\left(i\right)E\left(i\right)$$

What needs to be commented is that “n”, “Coef(i)”, E(i) represented the number of signature genes, the coefficient index, and the gene expression level, respectively.

**Table 2:** 33 pyroptosis-related genes

Gene	Full-name
AIM2	Absent in melanoma 2
CASP1	Cysteine-aspartic acid protease-1
CASP3	Cysteine-aspartic acid protease-3
CASP4	Cysteine-aspartic acid protease-4
CASP5	Cysteine-aspartic acid protease-5
CASP6	Cysteine-aspartic acid protease-6
CASP8	Cysteine-aspartic acid protease-8
CASP9	Cysteine-aspartic acid protease-9
ELANE	Elastase, neutrophil expressed
GPX4	Glutathione peroxidase 4
GSDMA	Gasdermin A
GSDMB	Gasdermin B
GSDMC	Gasdermin C
GSDMD	Gasdermin D
GSDME	Gasdermin E
IL18	Interleukin 18
IL1B	Interleukin 1 beta
IL6	Interleukin 6
NLRC4	NLR family CARD domain containing 4
NLRP1	NLR family pyrin domain containing 1
NLRP2	NLR family pyrin domain containing 2
NLRP3	NLR family pyrin domain containing 3
NLRP6	NLR family pyrin domain containing 6
NLRP7	NLR family pyrin domain containing 7
NOD1	Nucleotide binding oligomerization domain containing 1
NOD2	Nucleotide binding oligomerization domain containing 2
PJVK	Pejvakin/deafness, autosomal recessive 59
PLCG1	Phospholipase C gamma 1
PRKACA	Protein kinase cAMP-activated catalytic subunit alpha
PYCARD	PYD and CARD domain containing
SCAF11	SR-related CTD associated factor 11
TIRAP	TIR domain containing adaptor protein
TNF	Tumor necrosis factor

### Internal and external validation of models

The accuracy and specificity of the model were quantified by the area under the curve (AUC) of receiver operating characteristic (ROC), and then the influence of the included factors on the prognosis of patients was evaluated. According to the median risk score, patients in TCGA-PAAD and GEO cohort were divided into low-risk group and high-risk group. The survival curve between the risk score and OS of PDAC patients was plotted by Kaplan–Meier (K–M) method, and log-rank was used to test the significance of difference.

### Functional inference

Gene Set Enrichment Analysis (GSEA) is a software for gene sets enrichment (https://www.gsea-msigdb.org/gsea/index.jsp) [30, 31]. To clarify differences in gene function and signalling pathways in different prognosis of PDAC samples, we use GSEA (version 4.0.1) and “enrichplot” R package for enrichment analysis of Gene Ontology (GO) and Kyoto Encyclopedia of Gene and Genomics (KEGG).

### Methylation of CASP4 and landscape analysis of gene mutation

The human disease methylation database, DiseaseMeth version 2.0 is an interactive database focused on the aberrant DNA methylation in human diseases, especially various cancers. We used this website to analyze and compare the differences in CASP4 methylation between PDAC and para-cancer tissues and visualized them with box diagrams. R packet “maftools” was used to compare the frequency of individual gene mutations in TCGA-PAAD.

### Analysis of drug sensitivity related to PRGs

GSCALite (http://bioinfo.life.hust.edu.cn/web/GSCALite/) [32] is a comprehensive cancer analysis database that combines gene expression analysis, immunoinvasion analysis, mutation analysis and drug sensitivity analysis, containing 33 types of cancer from TCGA and Genomics of Drug Sensitivity in Cancer (GDSC). Through the “Drug Sensitivity Analysis” module of GDSC, we studied the correlation between PRGs and drug resistance in PDAC.

### Cell culture and transfection

Human pancreatic cancer cell line PANC-1 and Aspc-1, purchased from China Center for Type Culture Collection (CCTCC), was cultured with special medium (CM-0627, Procell Life Science & Technology Co., Ltd.) in the 37 °C cell incubator containing 5% CO_2_. When the cell density reached 50–70%, the cells were transfected with 20 nM siRNA (Table S1) and GenMute™ siRNA transfection reagent (SL100568, SignaGen Laboratories, USA).

### Real-time PCR

Following the manufacturer’s instructions, total RNA was extracted from cells with Trizol reagent (Invitrogen, Carlsbad, CA) and then measured using a NanoDrop ND-2000 spectrophotometer (Thermo Fisher Scientific). The RNA was reverse transcribed into cDNA using HiScript II Q RT SuperMix for qPCR (+gDNA Wiper) (R223-01, Vazyme, China). ChamQ Universal SYBR qPCR Master Mix (Q711-02, Vazyme, China) was used for qPCR. QPCR primers include CASP4, NLRP1, FASN, ACC, SREBP-1 and SREBP-2. The primer sequences used are listed in Table S2. Gene expression was normalized to the expression of GAPDH and calculated using 2^−ΔΔCT^ method. Three repeated experiments were set up in each group.

### Western blotting

48 h after siRNA transfection, the cells were washed twice with PBS. RIPA Lysis Buffer (BL504A, Biosharp, China) supplemented with protease and phosphatase inhibitors was added to the cells and lysed on ice for 15 min. The liquid was collected and centrifuged. Protein concentration was determined using BCA protein determination kit (P0012, Beyotime, China). Total protein was transferred to PVDF membrane (Millipore, Billerica, MA) after electrophoresis in 10% or 7.5% SDS-PAGE. After blocking the nonspecific sites on the membrane with 5% sealant for 1 h at room temperature, the membrane was applied to CASP4 (1:1000, #4450, Cell Signaling Technology, USA), NLRP1(1:1000, ab36852, abcam, USA), Vinculin (1:100000, V9264-100UL, Sigma-Aldrich), GSDMD (1:2000, TA4012, Abmart China), p-ERK (1:2000, #4370, Cell Signaling Technology, USA) and incubated overnight at 4 °C. Then, the membrane was incubated with HRP-Conjugated Affinipure Goat Anti-Rabbit IgG(H+L) (SA00001-2, Proteintech) at room temperature for 1 h. The blots were finally visualized with the ECL Chemiluminescence substrate (hypersensitive) (BL523B, Biosharp, China). The film was exposed and developed by X-ray in a darkroom. The film was scanned and the strips were and quantified by Image J (1.46R). Three repeated experiments were set up in each group.

### Cell proliferation assay

24 h after siRNA transfection, the cells were transfected into a 96-well culture plate (2000 cells/well). Following the manufacturer’s instructions, add 100 µL/well diluted 10 times CCK-8 reagent (CA1210, Beijing Solarbio Science & Technology Co., Ltd.) at 0, 24, 48, 72 h after laying plate, respectively. The cells were incubated at 37 °C for 2 h and the optical density (OD) at 450 nm was measured with a microplate reader. Three repeated experiments were set up in each group.

### Transwell invasion assay

Transparent PET Membrane 24 Well 8.0 μm tin (BD Biosciences, USA) was used for cell invasion ability detection. 24 h after siRNA transfection, 2 × 10^4^ cells were inoculated into the upper chamber, and 500 μL of complete medium was added into the lower chamber. After the cell morphology was restored, the cells were starved for 8 h. Then the medium containing 20%FBS was changed into the lower chamber to induce membrane penetration. 24 h later, it was fixed with 4% paraformaldehyde for 30 min and stained with crystal violet for 15 min. Images were taken under microscope and analyzed by Image J (1.46R). Three repeated experiments were set up in each group.

### BODIPY staining

BODIPY staining was used to observe the neutral lipid droplets in PANC-1 and Aspc-1 cells after treatment. The cells were inoculated on cover glass, and when the cell density reached 50–70%, they were cleaned with PBS for 3 times. The cells were fixed with 4% paraformaldehyde for 15 min and then cleaned with PBS again. Then, 2 μm BODIPY 493/503 (D3922, Thermo Scientific) was used to avoid light Staining for 30 min, and DAPI Staining Solution (C1005, Beyotime) was used to avoid light Staining for 5 min. After staining, soak with PBS for 3 times. Place the cover slide lightly on the slide dripping with antifade mounting medium (P0126, Beyotime) and observe under a fluorescence microscope (Olympus, BX53).

### Immunohistochemistry (IHC)

The fresh tissue collected during the operation was immersed in 4% paraformaldehyde and embedded in paraffin. The wax blocks were sectioned and dewaxed, followed by antigen repair with 3% H_2_O_2_ and citrate buffer. The samples were then incubated overnight in primary antibody, followed by secondary antibody and hematoxylin. The primary antibodies used in this study were: CASP4 (1:200, #4450, Cell Signaling Technology, USA), NLRP1(1:200, ab36852, abcam, USA) Finally, the slices were viewed under the microscope.

Human protein mapping (HPA) (https://www.proteinatlas.org/) [33–35] provides information on the tissue and cellular distribution of almost all proteins available to the human. In this database, researchers used transcriptomic and proteomic techniques to study protein expression in different human tissues and organs on RNA and protein levels. HPA database was used to analyze the protein expression of PRGs and the immunohistochemical staining images were also downloaded.

### Lactic dehydrogenase (LDH) release detection

In order to detect the occurrence of cell pyrosis, the LDH Release Assay Kit (C0016, Beyotime) was used to detect the activity of lactate dehydrogenase released during cytotoxicity according to manufacturer’s instructions and literature report [36, 37]. After transfection with siRNA, the cell supernatant was centrifuged and transferred to a 96-well plate. Add the LDH detection working solution and detect the absorbance at 490 nm after incubation at room temperature without light for 30 min, and LDH release (%) was calculated.

### Detection of drug sensitivity

To test whether the expression of CASP4 or NLRP1 affects the sensitivity of pancreatic cancer cells to MEK inhibitors, Trametinib (MedChemExpress, USA) was added to siRNA-transfected PANC-1 and Aspc-1 cells. According to the preliminary experimental results and literature description [38, 39], the final concentration of Trametinib was 10 µM/L, and the cell survival of Aspc-1 was detected in 48 h after treatment, while PANC-1 was detected in 72 h.

### Statistical analysis

**Fig. 1:**
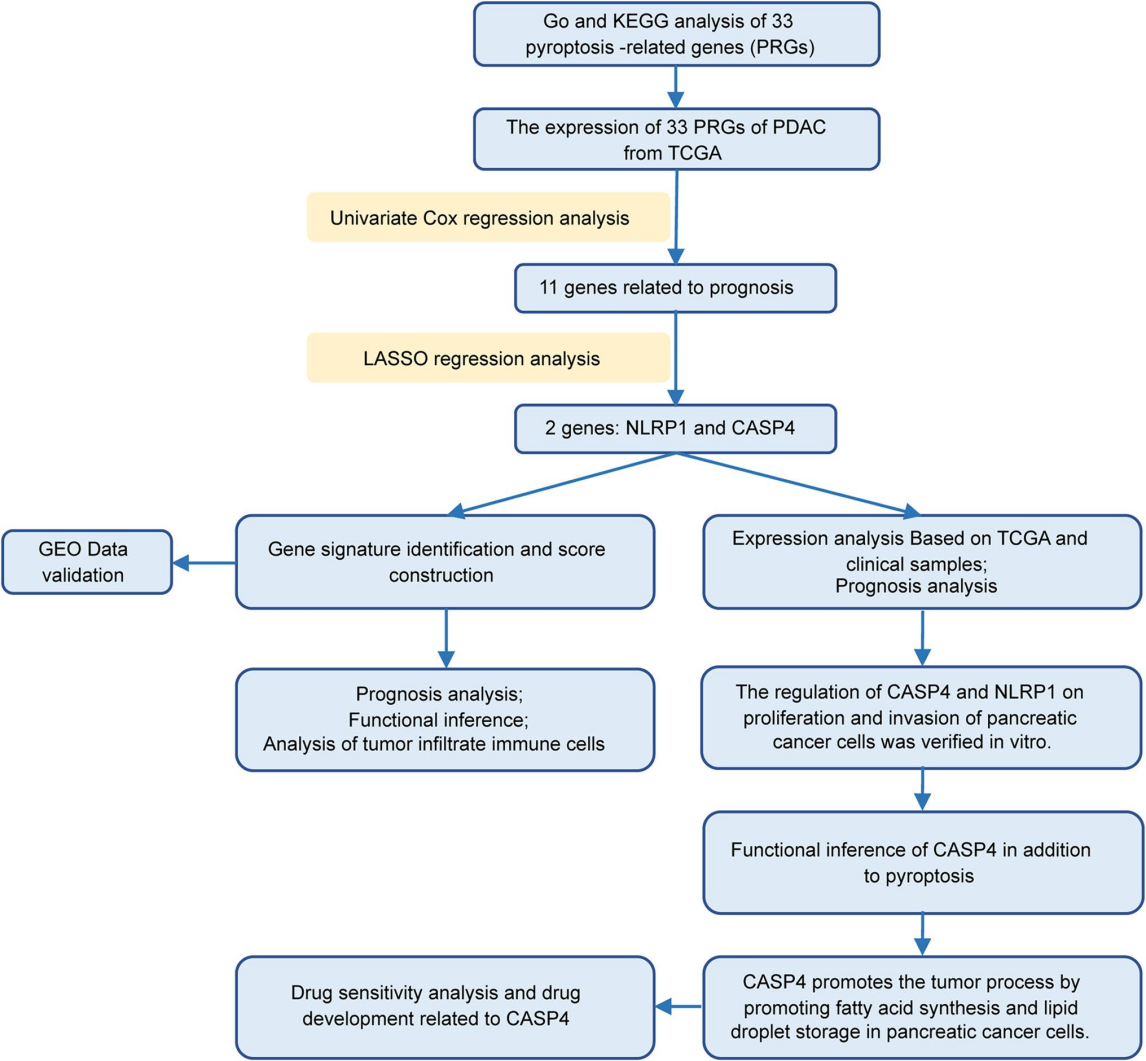
The flow chart of the present study

The research flow chart has been shown in Fig. 1. All survival curves are displayed with p-value from log-rank test. Mean and median for continuous variables were compared using independent t-test when the data were normally distributed; otherwise, the Mann–Whitney U test was used. Comparison of clinicopathological parameters and other classified variables was tested by chi-square test. The correlation of gene expression was evaluated by Spearman’s correlation and statistical significance. All tests were two-sided and P-value < 0.05 were considered statistically significant.

## Results

### Gene signature identification and risk scoring model construction

**Fig. 2:**
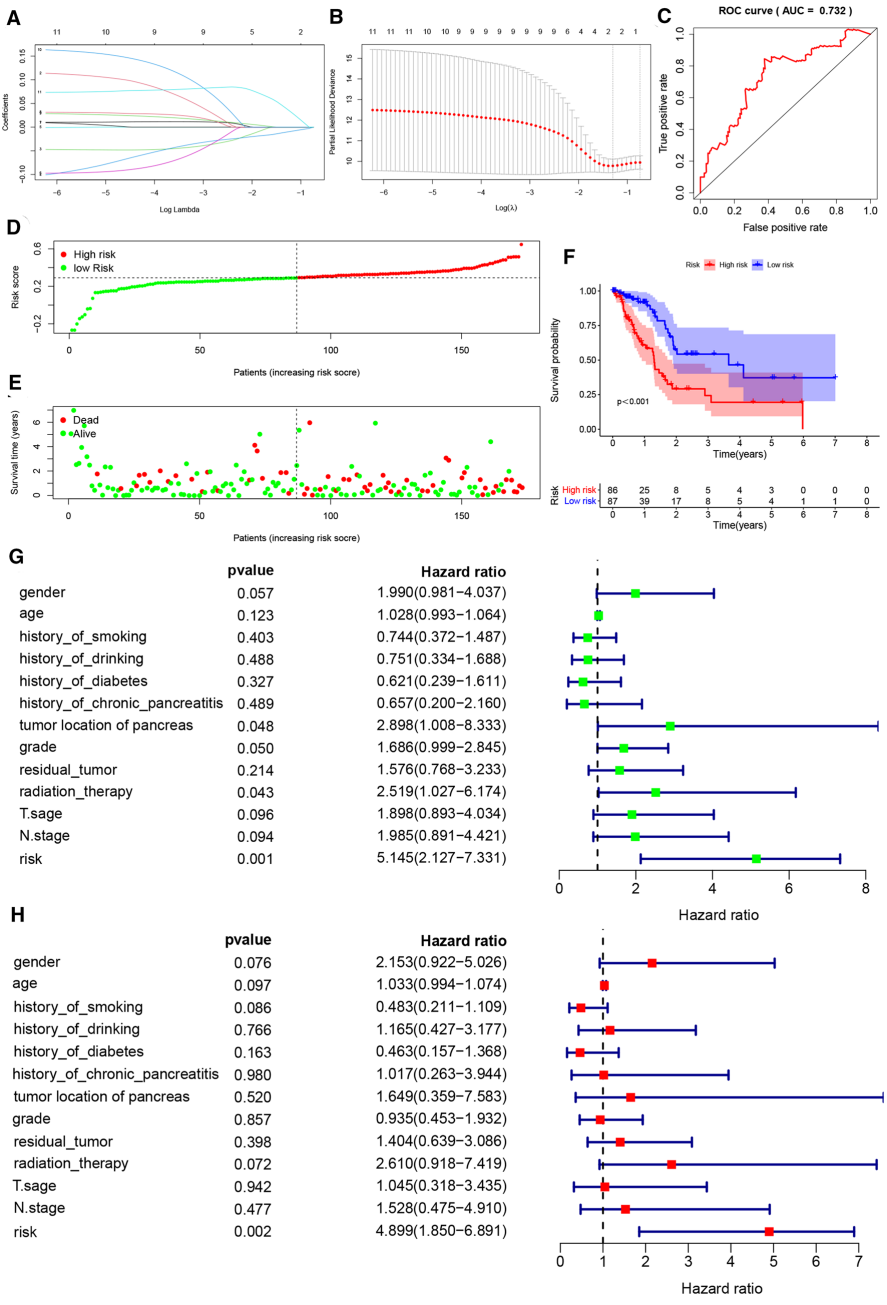
Identification of a 2-PRGs signature for PDAC patients and detection of predictive performance. **A** PRGs expression selection by using logistic regression with elastic net. Elastic net coefficient profiles of the 11 selected features. **B** LASSO Cox regression was used to select the most powerful parameter with cross-validation. **C** The ROC based on risk score. The risk score was divided into high-risk group and low-risk group with a cut-off value of 50%. **D**, **E** The distribution of risk score and survival status. **F** Kaplan–Meier survival analysis of PDAC patients in different risk groups from TCGA-PAAD cohort. **G** Tree diagram of a univariate regression analysis. **H** Tree diagram of a multivariate regression analysis. **P < 0.01, ***P < 0.001. Patients with tumors located in the body and tail of the pancreas received distal pancreatectomy, and patients with tumors located in the head of the pancreas received Whipple surgery

The selected PRGs (Table 2) were used for GO and KEGG analysis, and the results showed that these genes regulated multiple IL-1β related pathways (Figure S1). Firstly, a univariate Cox regression analysis was used to identify 11 PRGs associated with OS in TCGA-PAAD patients (P < 0.05) (Table 3). To identify the most powerful prognostic gene markers, Elastic net regularization and LASSO regression analysis was used to screen two PRGs and construct a prognostic gene signature (Fig. 2A, B), thus minimizing the risk of overfitting. Using cross-validation and minimization of the averaged error curves, we selected an optimal tuning parameter λ of 0.2733 with log (λ) of − 0.6. The risk score of patients was calculated based on the expression level and regression coefficient, which was as follows: Risk score = − 0.0105613982870017 * (the expression of NLRP1) + 0.0393442154637877 * (the expression of CASP4). Figure S2A displayed the expression levels of CASP4 and NLRP1 in tumour samples with different T stage and lymph node status. To further evaluate the impact of risk score on the prognosis of PDAC patients, K–M analysis showed that the prognosis of high-risk group (n = 86) was significantly poorer than that of low-risk group (n = 87) (P < 0.001) (Fig. 2F). In addition, we also compared the distribution of T stage and lymph node status among patients with different scores, showing that patients in the high-risk group had higher T stage and more lymph node metastases (Figure S2B). The distribution of survival status and risk scores of patients was shown in Fig. 2D, E, indicating that by the time of follow-up, more PDAC patients had died in the high-risk group than in the low-risk group. The ROC curve showed good predictive power of the model for predicting the prognosis of PDAC patients based on the gene signature (AUC = 0.732, P < 0.001) (Fig. 2C).

**Table 3:** Tree diagram of univariate Cox regression between PRGs and prognosis of PDAC

ID	HR	HR.95L	HR.95H	p-value
NLRP2	1.080016	1.022584	1.140673	0.005762
CASP8	1.269678	1.09664	1.470019	0.001403
PRKACA	0.89609	0.83489	0.961776	0.002368
NLRP1	0.873237	0.784275	0.97229	0.013415
PYCARD	1.023932	1.004007	1.044253	0.018333
PLCG1	0.87138	0.778094	0.975849	0.017166
CASP1	1.081328	1.006057	1.16223	0.033669
GSDMC	1.103778	1.011484	1.204493	0.026673
IL18	1.050832	1.023062	1.079355	0.000285
AIM2	1.105527	1.020615	1.197503	0.013878
CASP4	1.204421	1.110487	1.306301	7.14E−06

In order to further analyse the prognostic value of PRGs characteristics in PDAC patients, univariate and multivariate Cox regression analyses were performed on clinicopathological characteristics, including age at diagnosis, sex, smoking history, drinking history, diabetes history, history of chronic pancreatitis, tumor site, histological grade, T stage, N stage, residual tumor and radiotherapy, and risk score. The results indicated that risk score based on PRGs signature was an independent prognostic factor for PDAC patients (P = 0.002, HR = 4.899, 95% CI 1.850–6.891) (Fig. 2G, H).

### External validation of the gene signature

**Fig. 3:**
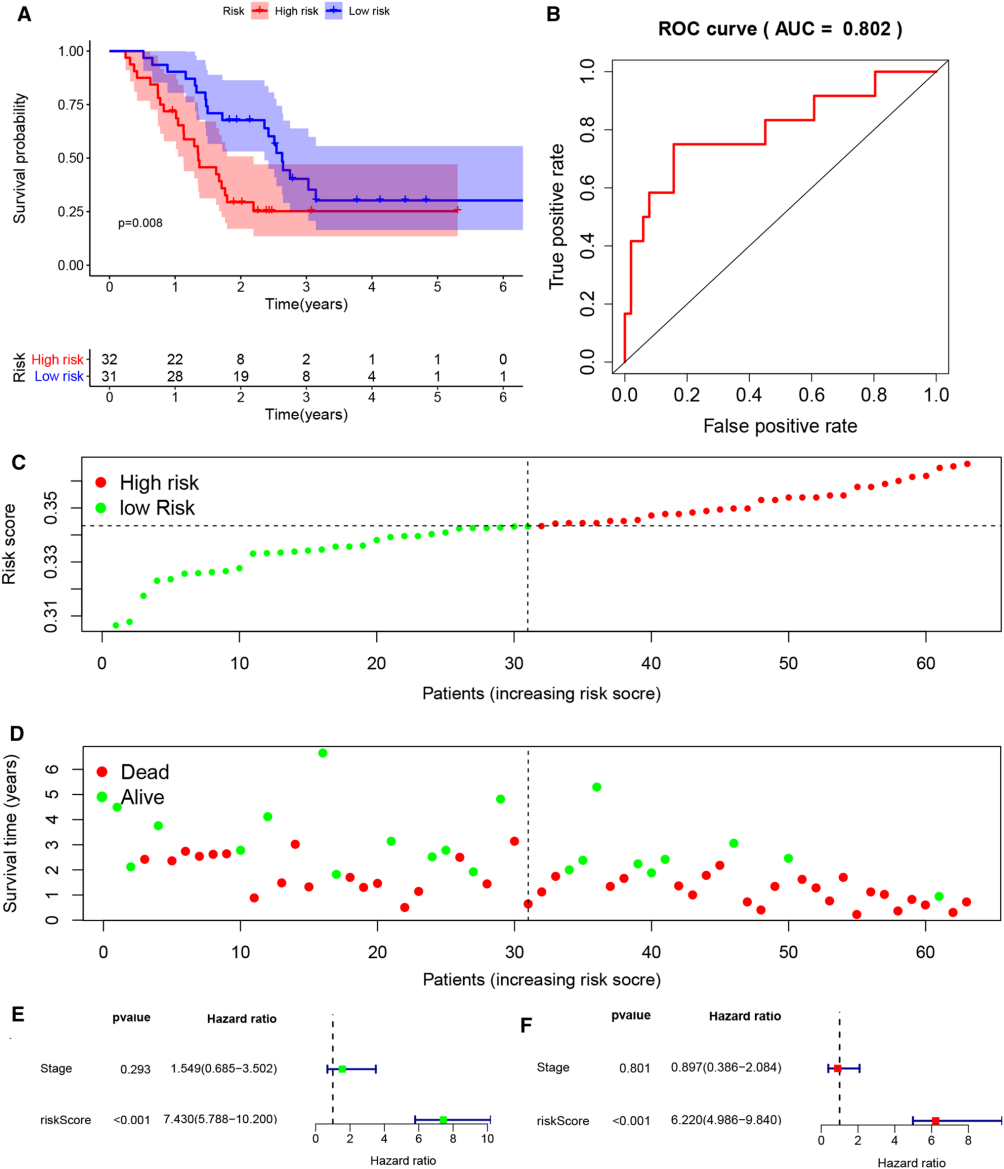
External validation of the risk prediction model using GSE57495 dataset. **A** Kaplan–Meier survival analysis of PDAC patients in the high-risk group and low-risk group. **B** The ROC based on risk score. **C**, **D** The distribution of risk score and survival status. **E** Tree diagram of a univariate regression analysis. **F** Tree diagram of a multivariate regression analysis

**Fig. 4:**
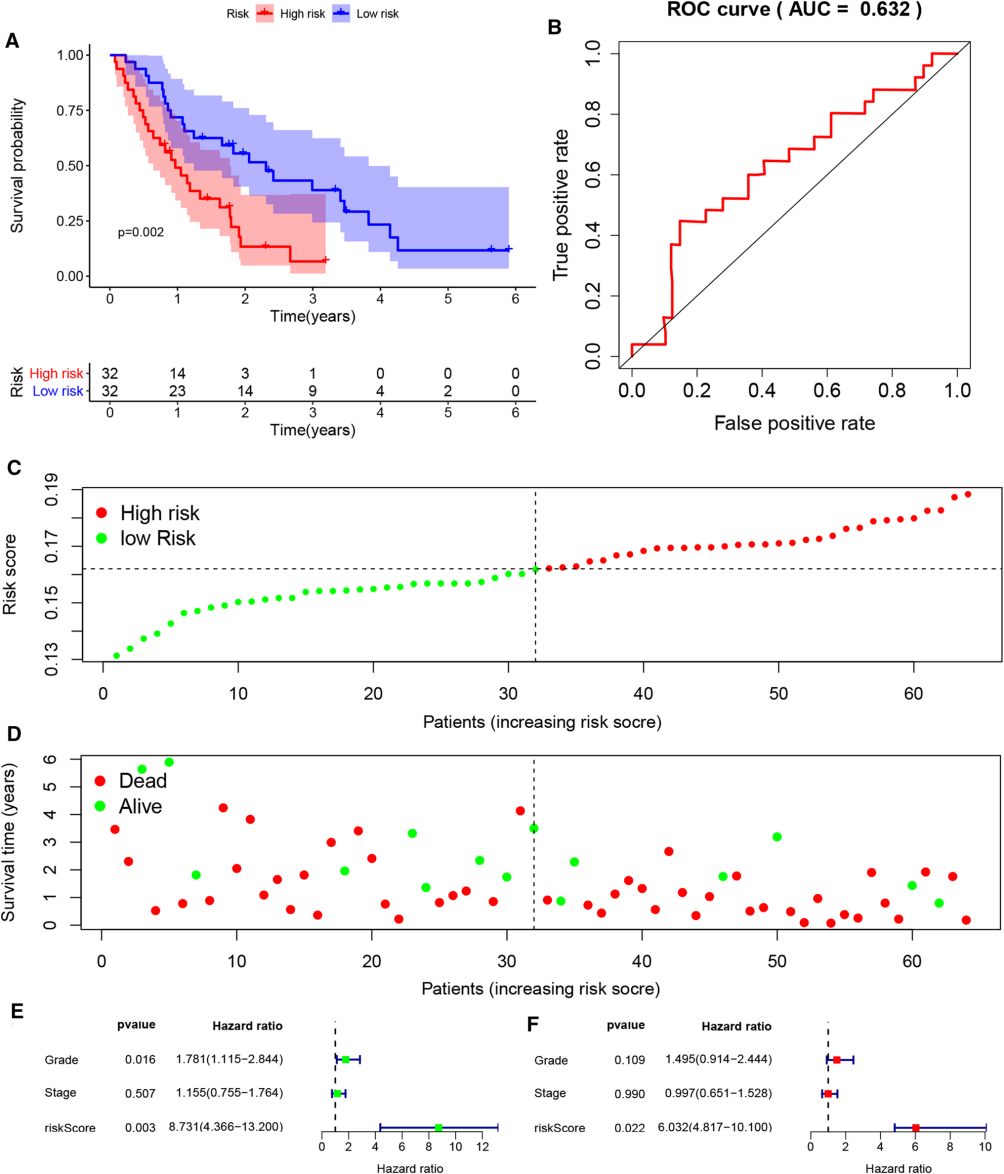
External validation of the risk prediction model using GSE62452 dataset. **A** Kaplan–Meier survival analysis of PDAC patients in the high-risk group and low-risk group. **B** The ROC based on risk score. **C**, **D** The distribution of risk score and survival status. **E** Tree diagram of a univariate regression analysis. **F** Tree diagram of a multivariate regression analysis

In order to further verify the effect value of the gene signature based on PRGs, we downloaded two datasets, GSE57495 and GSE62452 from GEO. The expression levels of CASP4 and NLRP1 in tumour samples with different stage and grade was shown in Figure S2E, F, which could roughly show that the higher the tumour stage and grade, the higher the expression level of CASP4, while NLRP1 is the opposite. The risk score of the sample of data set was calculated according to the formula, and the patients were also divided into low-risk group and high-risk group. K–M analysis showed that the prognosis of the high-risk group was significantly worse than that of the low-risk group (P = 0.008, P = 0.002) (Figs. 3A and 4A). Besides, as we can see from Figure S2C, D, the predicted high-risk group actually contained more patients with higher grade and higher stage. More PDAC patients in the high-risk group had died by the time of follow-up and had survived less than those in the low-risk group (Figs. 3C, D and 4C, D). The ROC curve showed that the model had good predictive ability (AUC = 0.802, AUC = 0.632, P < 0.05) (Figs. 3B and 4B). Although the sample size was limited, the risk score based on PRG can still be used as an independent prognostic factor for OS of PDAC patients (P < 0.05), while stage and grade cannot (Figs. 3E, F and 4E, F).

### Expression and survival analysis of PRGs

**Fig. 5:**
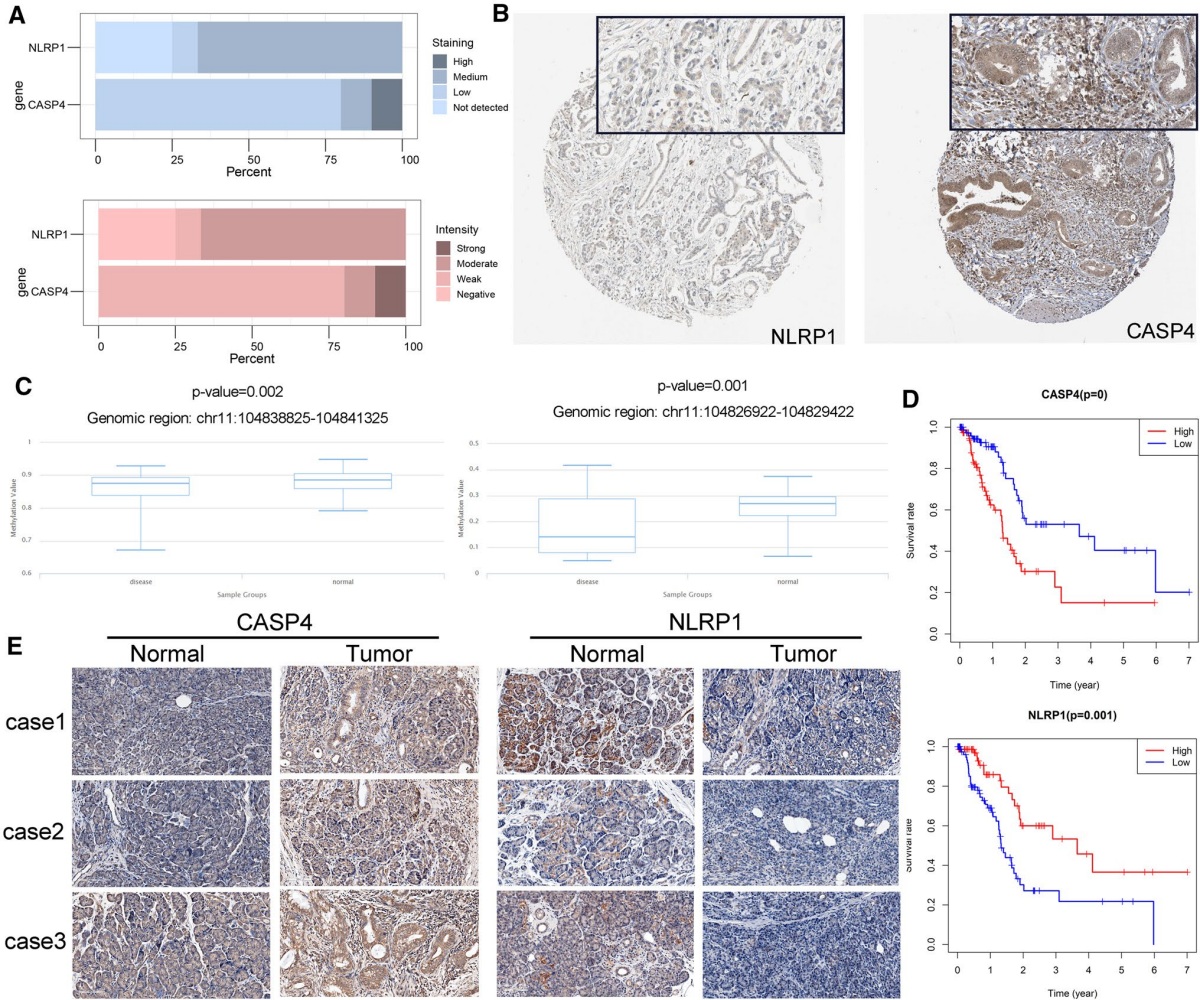
Expression and survival analysis of PRGs in PDAC. **A** The protein expression score of staining and intensity of NLRP1 and CASP4 proteins in all PDAC samples (HPA database). Protein expression score is based on immunohistochemical data manually scored with regard to staining intensity (negative, weak, moderate or strong) and fraction of stained cells (< 25%, 25–75% or > 75%). **B** Immunohistochemical staining of NLRP1 and CASP4 proteins in PDAC. **C** Methylation levels of CASP4 at chr11:104838825–104841325 and chr11:104826922–104829422 in PDAC and para-cancer tissues. **D** The OS survival curves of NLRP1 and CASP4 mRNA expression level in PDAC. **E** CASP4 and NLRP1 immunohistochemistry for patients from Zhongnan Hospital (×200)

Figure 5A, B, exhibiting the immunohistochemical staining and intensity of NLRP1 and CASP4 proteins in all PDAC samples, showed that NLRP1 staining was weak, while the protein expression level of CASP4 were elevated in PDAC samples. To further analyse the reasons for the difference in CASP4 expression between cancer and para-cancer, we used DiseaseMeth database to compare the methylation level of CASP4. The results showed that CASP4 was located at two sites on the chromosome in which the mean methylation level of CASP4 in PDAC was significantly lower than in para-cancer tissues (P < 0.05) (Fig. 5C). Besides, the survival curve showed that high expression of NLRP1 and low expression of CASP4 were associated with a better prognosis (P < 0.05) (Fig. 5D). Immunohistochemistry indicated that CASP4 was expressed at high levels in tumor tissues but weakly expressed in adjacent normal tissues, and the difference in NLRP1 expression between tumor and adjacent normal tissues was opposite to that of CASP4 (Fig. 5E).

### PRGs regulate the proliferation and invasion of pancreatic cancer cells in vitro

**Fig. 6:**
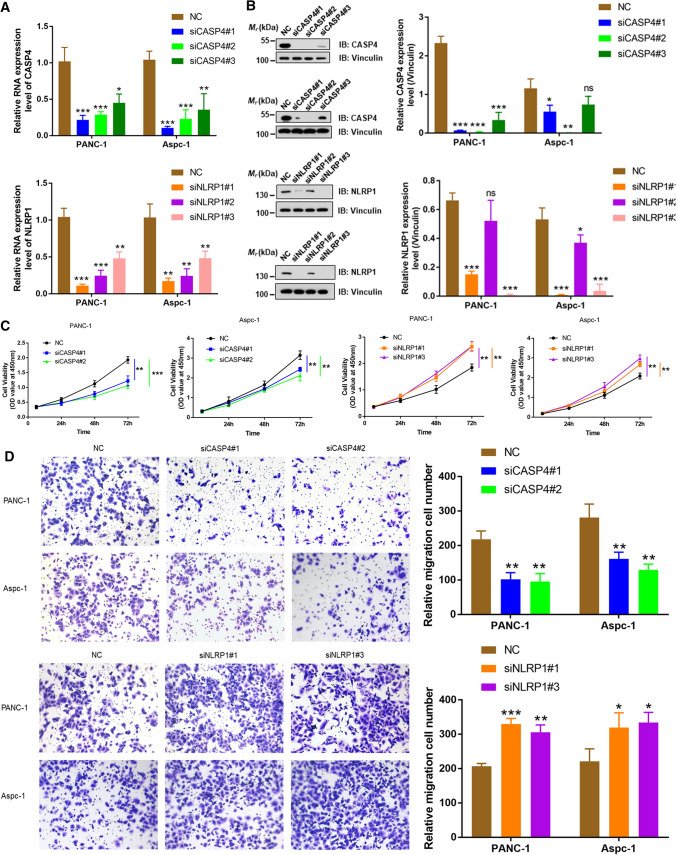
PRGs regulated the proliferation and invasion of pancreatic cancer cells in vitro. **A**, **B** After siRNA transfection, qPCR and western blot was used to detect RNA and protein expression levels of CASP4 and NLRP1 respectively in PANC-1 and Aspc-1 cells. **C** CCK8 assay was used to detect the proliferation of PANC-1 and Aspc-1 cells after transfection with siRNA. **D** Transwell was used to detect the change of cell invasion ability after transfection with siRNA (*P < 0.05, **P < 0.01, ***P < 0.001)

To further evaluate the functional significance of CASP4 and NLRP1 in PDAC, we conducted an in vitro study to examine the effects of CASP4 and NLRP1 on PANC-1 and Aspc-1. First, RNA and protein expression levels of CASP4 and NLRP1 in cells were knocked down by transfection of siCASP4#1, siCASP4#2, siNLRP1#1 and siNLRP1#3 (P < 0.01), which were for all subsequent experiments (Fig. 6A, B). CCK-8 showed that CASP4 knockdown significantly inhibited the cell viability of PANC-1 and Aspc-1, while NLRP1 knockdown significantly enhanced the cell viability (P < 0.01) (Fig. 6C). In addition, Transwell results showed that knocking down CASP4 significantly inhibited the invasion and migration of PDAC cells, which were promoted by knocking down NLRP1 (P < 0.05) (Fig. 6D). These results suggested that CASP4 may accelerate the progression of PDAC by promoting proliferation, invasion and migration of pancreatic cancer cells, while NLRP1 has been found to have tumor suppressive effect in vitro.

### CASP4 could regulate accumulation of lipid droplets

**Fig. 7:**
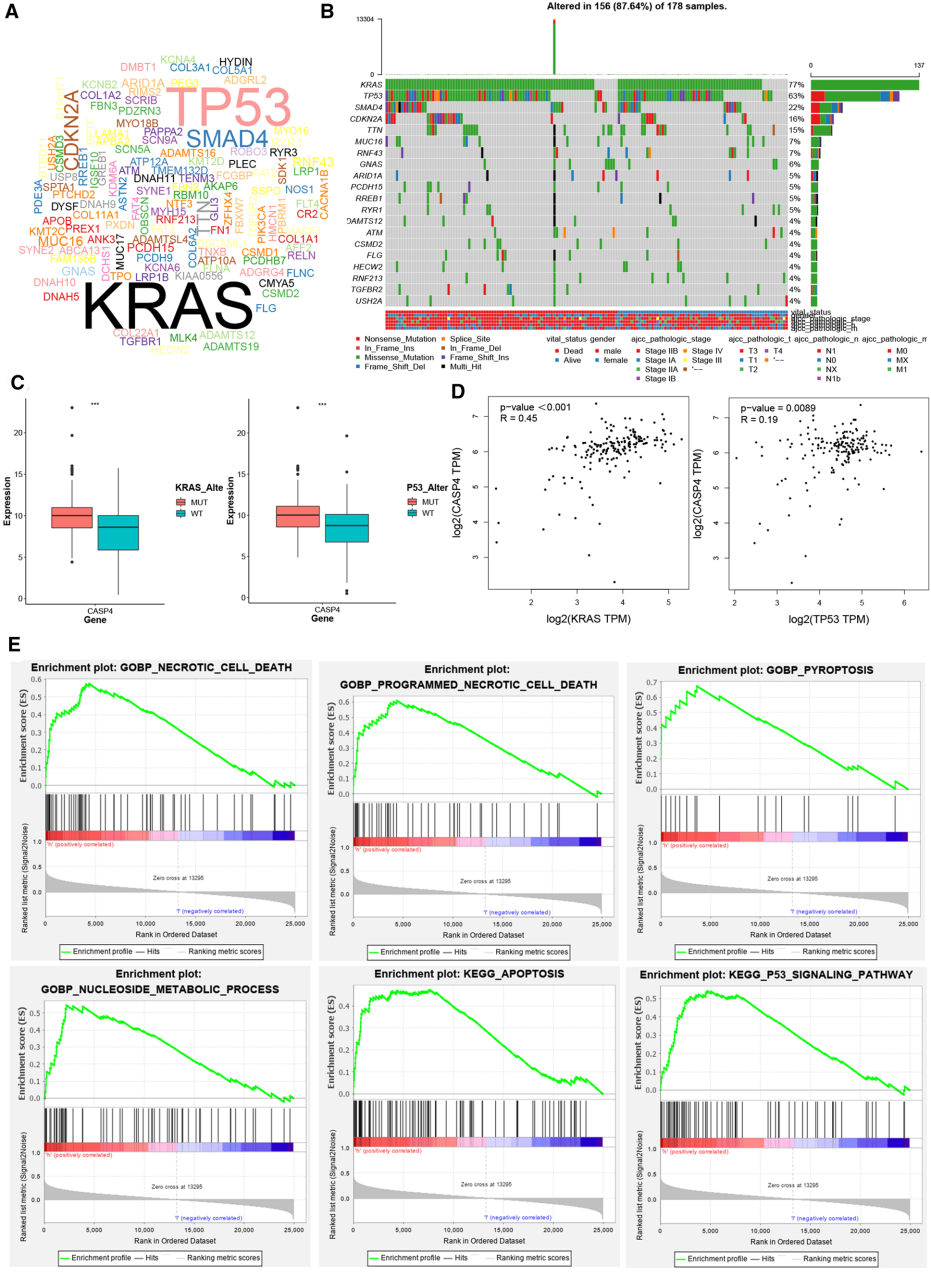
CASP4 was associated with KRAS and P53 mutations potentially. **A**, **B** Cloud plot and waterfall plot of mutant landscape in TCGA-PAAD samples. **C** The expression levels of CASP4 were different in KRAS mutation, P53 mutation and wild-type tumor tissues respectively. **D** Correlation curve between CASP4, KRAS and P53 expression levels based on TCGA database. **E** GSEA analysis of DEGs in high CASP4 expression group. ***P < 0.001

KRAS and P53 mutations are most common in pancreatic cancer (Fig. 7A, B). Based on this, PDAC samples were grouped according to KRAS and P53 mutations, and we compared the expression level of CASP4 in mutant and wild-type tumors. We found that the expression level of CASP4 was higher in both KRAS mutated samples and P53 mutated samples than in wild-type samples (P < 0.001) (Fig. 7C). Meanwhile, correlation analysis also showed that CASP4 was significantly positively correlated with KRAS and P53 expression level respectively (R = 0.45, R = 0.19, P < 0.001) (Fig. 7D). In addition, gene sets enrichment analysis also suggested that the differentially expressed genes (DEGs) in CASP4 high expression samples were mainly involved in regulating programmed cell death, nucleotide metabolism and P53 signaling pathway (Fig. 7E).

**Fig. 8:**
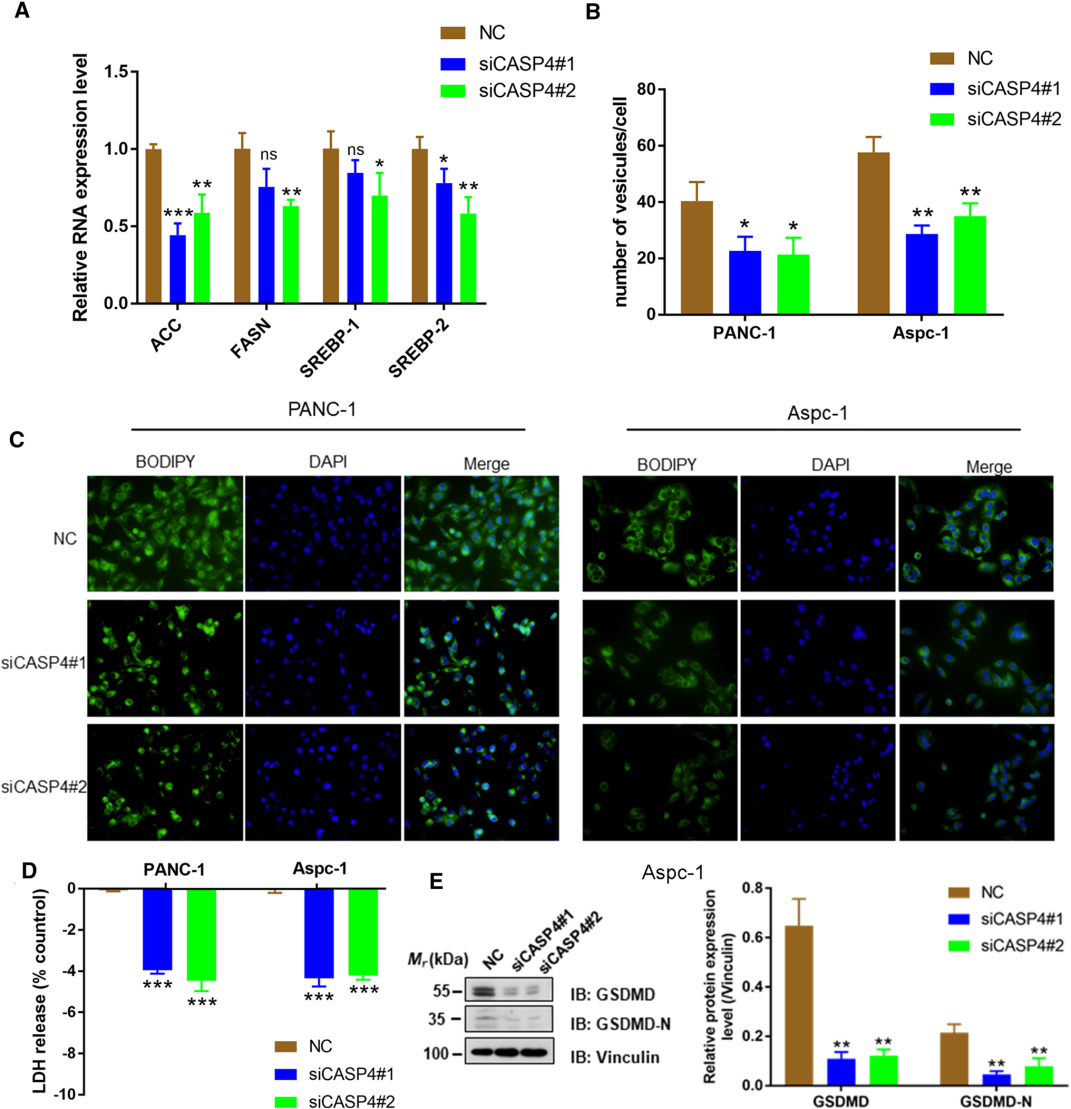
CASP4 promoted tumor progression by regulating accumulation of lipid droplets. **A** RNA expression level of key enzyme molecules in fatty acid synthesis after transfecting shCASP4 in PANC-1 and Aspc-1 cells. **B**, **C** The mean number of lipid vesicles per cell. The lipid droplets were counted randomly (≥ 50 cells were counted per condition). **D** PANC-1 and Aspc-1 cells were tested for LDH release 24 h after siCASP4 transfection. **E** Cleavage of GSDMD were monitored by immunoblot analysis 48 h after siCASP4 transfection (*P < 0.05, **P < 0.01, ***P < 0.001)

To further explore the mechanism of CASP4 promoting cancer, we knocked down CASP4 by transfection with shRNA in PANC-1 and Aspc-1 cells, and then detected the expression of key enzyme molecules in fatty acid synthesis. The results showed that low CASP4 expression significantly reduced the RNA levels of acetyl-CoA carboxylase (ACC), FASN, SREBP-1 and SREBP-2 (P < 0.05) (Fig. 8A). In addition, immunofluorescent staining displayed that the number of lipid droplets decreased significantly after CASP4 knockdown (P < 0.05) (Fig. 8B, C). Meanwhile, knockdown of CASP4 caused a decrease % of LDH released and GSDMD-N in cells (P < 0.01), suggesting that the degree of cell pyroptosis was reduced (Fig. 8D, E).

### NLRP1 was involved in the regulation of RAS/ERK signaling in pancreatic cancer

**Fig. 9:**
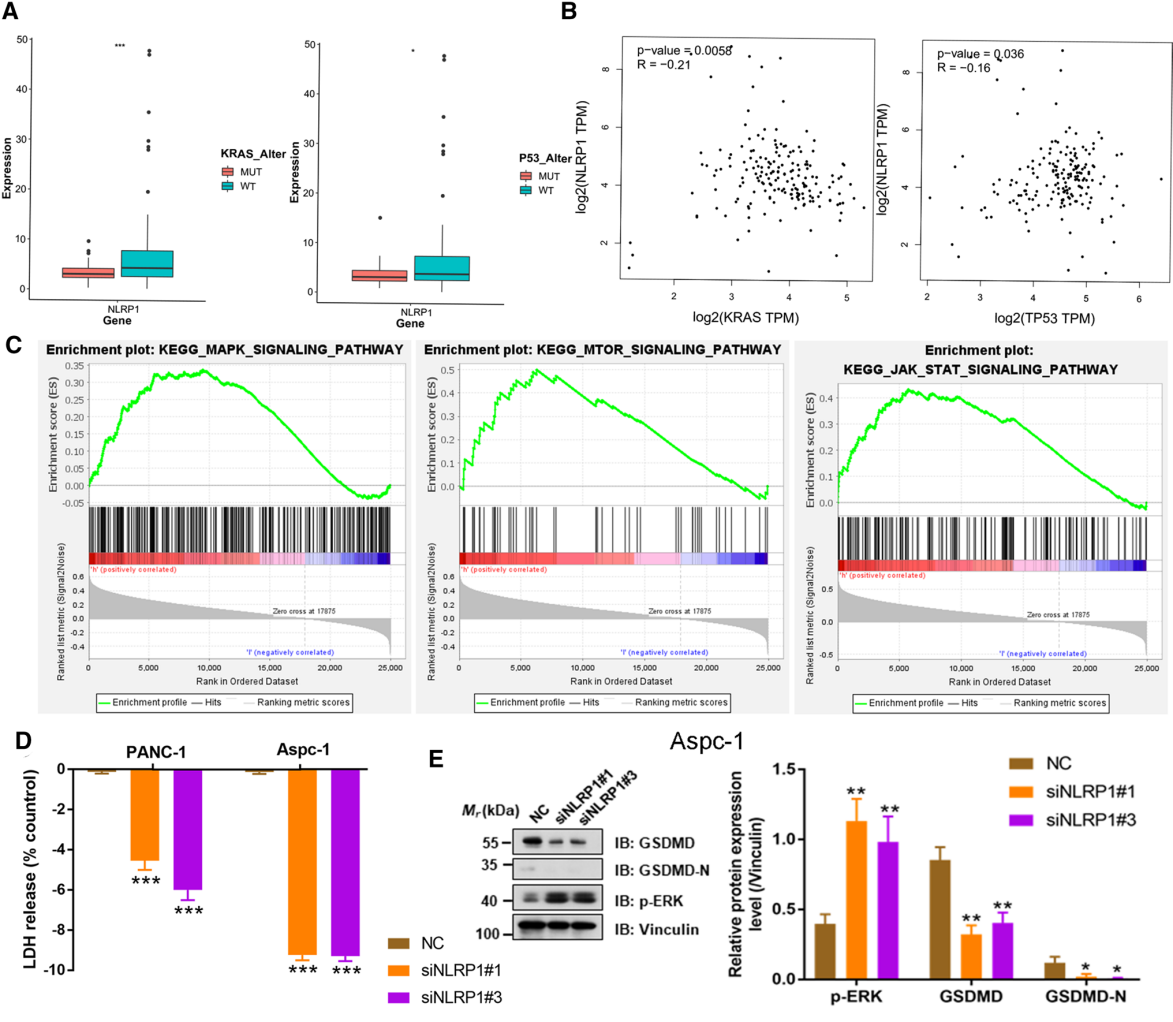
NLRP1 was associated with KRAS and P53 mutations potentially. **A** The expression levels of NLRP1 were different in KRAS mutation, P53 mutation and wild-type tumor tissues respectively. **B** Correlation curve between NLRP1, KRAS and P53 expression levels based on TCGA database. **C** GSEA analysis of DEGs in high NLRP1 expression group. **D** PANC-1 and Aspc-1 cells were tested for LDH release 24 h after siNLRP1 transfection. **E** Cleavage of GSDMD and p-ERK were monitored by immunoblot analysis 48 h after siNLRP1 transfection (*P < 0.05, ***P < 0.001)

To investigate the mechanism of NLRP1 involved in the regulation of pancreatic cancer progression, the same analysis was performed for NLRP1 to test whether NLRP1 was associated with KRAS mutation. The results showed that NLRP1 expression level was significantly decreased in pancreatic cancer samples with KRAS or P53 mutations (P < 0.05) (Fig. 9A). Moreover, NLRP1 was significantly negatively correlated with KRAS or P53 (R = − 0.21, R = − 0.16, P < 0.05) (Fig. 9B). Gene set enrichment analysis also showed that NLRP1 may be involved in the regulation of MAPK, mTOR and JAK/STAT signaling pathways (Fig. 9C). We further verified this result in vitro and found that after siNLRP1 knockdown, the expression level of p-ERK in cells was significantly increased (Fig. 9E). Meanwhile, knockdown of NLRP1 caused a decrease % of LDH released and GSDMD-N in cells (P < 0.05), suggesting that the degree of cell pyroptosis was reduced (Fig. 9D, E). However, knocking down NLRP1 did not significantly affect the number of lipid droplets in either type of cell (P > 0.05) (Figure S3).

### Analysis of drug sensitivity of PRGs

**Fig. 10:**
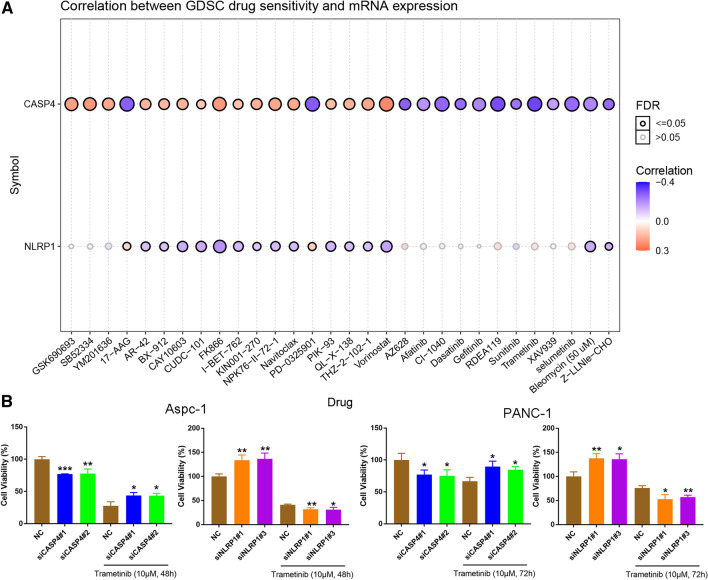
Analysis of drug sensitivity associated with PEGs. **A** Correlation of CASP4 and NLRP1 expression with multidrug sensitivity in GDSC. Red is positive correlation, which means the higher the gene expression, the more sensitive to the drug, while the blue is the opposite. The Spearman correlation represent the gene expression correlates with the drug. The positive correlation means that the gene high expression is resistant to the drug, vise verse. **B** Survival rate of cells exposed to trametinib at the concentrations of 10 µM/L for 48 or 72 h (*P < 0.05, **P < 0.01, ***P < 0.001)

Figure 10A, displaying the correlation analysis between PRGs and drug sensitivity in PDAC, showed that CASP4 was significantly positively related with FK866, the inhibitor of nicotinamide phosphoribosyltransferase and vorinostat, the inhibitor of histone deacetylase inhibitors of sensitivity, and NLRP1 negatively correlated with them. In addition, CASP4 was negatively correlated with 17-AAG, the inhibitor of AKT, Mirdametinib (PD-0325901), Refametinib (RDEA-119), Cl-1040, and Trametinib, the inhibitors of MEK. Trametinib was used to treat siCASP4-transfected Aspc-1 and PANC-1, and the cell viability was significantly increased compared with the control group 48 or 72 h later, while the viability of siNLRP1 transfected cells was significantly decreased (P < 0.05) (Fig. 10B). These results suggested that up-regulation of CASP4 or inhibition of NLRP1 expression can enhance the sensitivity of pancreatic cancer cells to trametinib.

## Discussion

PDAC is one of the most common and deadly solid tumors. Despite significant advances in diagnosis and treatment, the clinical outcome of PDAC is still poor, mainly due to low surgical resection rate and postoperative recurrence. Other therapeutic strategies, such as combination chemotherapy, molecularly targeted agents, and immune checkpoint inhibitors, have limited efficacy due to tumor heterogeneity and inherent resistance [40, 41]. Tumorigenesis is associated with a variety of factors, including activation of proto-oncogenes and anticancer genes, TME, oxidative stress, and chronic inflammatory stimuli. Activation of pyroptosis leads to the release of the inflammatory mediators IL-1 and IL-18, which can contribute to the development of cancer in a number of ways. For another, pyroptosis can promote tumor cell death, making it a potential prognostic marker and therapeutic target for cancer. Therefore, PRGs play different roles in the occurrence and progression of different cancers. For example, pyroptosis inhibits the progression of hepatocellular carcinoma, colorectal cancer and gastric cancer [42–47], but it promotes the proliferation and metastasis of breast cancer cells [48]. However, the role of PRGs in PDAC has not been clarified. Therefore, in this study, we aimed to discover a novel prognostic marker and therapeutic target related to pyroptosis through data analysis and mechanism exploration to provide potential approaches in the treatment of PDAC.

In order to further evaluate the prognostic value of these PRGs, we constructed a risk score model based on NLRP1 and CASP4 gene signature through univariate Cox analysis and LASSO regression analysis, and then verified their good predictive performance in external datasets. Previous studies have shown that NLRP1 is considered a tumor suppressor gene. NLRP1 is one of inflammasome sensors, the activator of which induces the proteasome-mediated destruction of the N-terminal fragment and liberates the C-terminal fragment to form an inflammasome [49]. Inflammasome represents a group of protein complexes that induce inflammation and pyroptosis, and its abnormal and chronic activation is the pathological basis for many common inflammatory diseases and tumorigenesis [50]. Targeting the activation of NLRP1 in epidermal keratinocytes represented a potential therapeutic strategy for NLRP1-dependent inflammatory skin disease and cancer [51, 52]. In our study, NLRP1 was believed to inhibit the occurrence and progression of PDAC, which may be due to NLRP1’s involvement in the regulation of MAPK, mTOR and JAK/STAT signaling pathways. Notably, NLRP1 was negatively correlated with KRAS, and inhibition of NLRP1 expression enhanced the effect of ERK inhibitors, which was potentially valuable for the treatment of PDAC. There have been few studies on the regulation of NLRP1 and RAS/ERK signaling pathways in tumors. Zhai et al. [53] found that NLRP1 functioned downstream of the MAPK/ERK signaling and contributed to acquired targeted therapy resistance in human metastatic melanoma, which was consistent with our findings.

The prognostic significance of CASP4 overexpression in cancers remains controversial. For example, the clinical cohort study of Shibamoto et al. showed that CASP4 may play a role as a tumor suppressor gene in esophageal cancer and as a potential biomarker for predicting esophageal cancer prognosis [54, 55]. However, silencing CASP4 gene inhibited the migration, adhesion, and invasion of epithelial cancer cells [56]. Meng et al. found that CASP4 was highly expressed in renal clear cell carcinoma based on TCGA data, suggesting poor prognosis, and was associated with tumor drug resistance [57]. In our study, CCK8 and transwell assay suggested that CASP4 may accelerate the progression of PDAC by promoting proliferation and migration of pancreatic cancer cells. It is noteworthy that CASP4 is commonly known as a cell pyroptosis gene, but it has been found to promote cancer in some experimental and clinical studies, the mechanism of which has not been explored. KRAS and P53 mutations, the most common mutation in pancreatic cancer, can change normal metabolic pathways and initiate metabolic reprogramming by activating transcription factors and enhancing enzyme activity [58, 59]. By grouping pancreatic cancer samples in TCGA, we found that the expression level of CASP4 was higher in both KRAS and P53 mutation samples than in wild-type samples. In addition, Michela Terlizzi analyzed changes in lipid metabolism characteristics in CASP4-positive non-small cell lung cancer and found increased palmitic acid and malonic acid in tissues of CASP4-positive patients, which are important for fatty acid biosynthesis and elongation [60, 61]. Therefore, it is reasonable to speculate that CASP4 may be one of the factors in the synergistic regulatory network of KRAS and P53 and promoted the biosynthesis of fatty acids in pancreatic cancer and reserves productive substrates for the proliferation and migration of tumor cells in addition to the occurrence of pyroptosis. Our experiment results suggested that CASP4 knockdown in PANC-1 and Aspc-1 cells significantly reduced the number of lipid droplets, and the expression of key enzymes and transcription factors involved in fatty acid synthesis, which was the first in vitro study of CASP4 regulation of pancreatic cancer lipid metabolism.

We tried to apply the results of this study into clinical practice, not only establishing a prognostic risk model, but also exploring the correlation between PRGs and tumor drug resistance. Through drug sensitivity analysis, CASP4 and NLRP1 were significantly related with the inhibitors of AKT and MEK and elevated CASP4 or inhibited NLRP1 may enhanced efficiency of trametinib as the CCK8 results suggested. But it was the limitation of this study that we haven’t conducted experiments to investigate whether the expression of CASP4/NLRP1 influenced the sensitivity of pancreatic cancer to these drugs., such as comparing tumor growth by using MEK or AKT inhibitors in vivo with animals. As a continuation of future research, we will supplement it in future research. Besides, because this is a retrospective study, we call for a prospective study with a larger sample size to verify the clinical application of PRGs in personalized management of PDAC patients.

## Conclusions

In this study, we used TCGA-PAAD RNA-seq data and clinical data to construct a risk prediction model based on PRGs, including NLRP1 and CASP4. In addition to pyroptosis, CASP4 may promote pancreatic cancer cell migration by promoting fatty acid synthesis, as well as NLRP1 was also closely related to the RAS/ERK signaling pathway. CASP4 and NLRP1 are expected to be important prognostic markers and therapeutic targets for PDAC, and corresponding targeted drugs are emerging.

## Supplementary Information

Below is the link to the electronic supplementary material.

Supplementary file 1 (DOCX 1933.8 kb)

## Data Availability

The datasets generated and/or analysed during the current study are available in the TCGA (https://portal.gdc.cancer.gov/) and GEO (https://www.ncbi.nlm.nih.gov/geo/) (GSE62452; GSE57495). The data that support the findings of this study are available from the corresponding author upon reasonable request.

## Abbreviations

PDAC: Pancreatic ductal adenocarcinoma
PRGs: Pyroptosis-related genes
TCGA: The Cancer Genome Atlas
TME: Tumor microenvironment
LDH: Lactic dehydrogenase
GSDM: Gasdermin
GEO: Gene Expression Omnibus
OS: Overall survival
LASSO: Least absolute shrinkage and selection operator
AUC: Area under the curve
ROC: Receiver operating characteristic
K–M: Kaplan–Meier
GSEA: Gene Set Enrichment Analysis
GO: Gene Ontology
KEGG: Kyoto Encyclopedia of Gene and Genomics
IHC: Immunohistochemistry
HPA: Human protein mapping
GDSC: Genomics of Drug Sensitivity in Cancer
CCTCC: China Center for Type Culture Collection
DEGs: Differentially expressed genes
OD: Optical density
