# Supplementary material for: Pyroptosis-related genes regulate proliferation and invasion of pancreatic cancer and serve as the prognostic signature for modeling patient survival
Source: Discov Oncol. 2022 May 28;13:39. doi: 10.1007/s12672-022-00495-0 (PMC9148360; doi:10.1007/s12672-022-00495-0)

**Supplementary Table. 1** Nucleotide sequence of siRNA

| Name | Sequences |
| --- | --- |
| siCASP4#1 | 5'-GGGUCUGGACUAUAGUGUATT-3' |
|  | 5'-UACACUAUAGUCCAGACCCTT-3' |
| siCASP4#2 | 5'-GAGACUAUGUAAAGAAAGATT-3' |
|  | 5'-UCUUUCUUUACAUAGUCUCTT-3' |
| siCASP4#3 | 5'-GCAAUCAUUUGAAACUCCATT-3' |
|  | 5'-UGGAGUUUCAAAUGAUUGCTT-3' |
| siNLRP1#1 | 5'-CGGUGACCGUUGAGAUUGATT-3' |
|  | 5'-UCAAUCUCAACGGUCACCGTT-3' |
| siNLRP1#2 | 5'-GGUGGAGCUGCAUCACAUATT-3' |
|  | 5'-UAUGUGAUGCAGCUCCACCTT-3' |
| siNLRP1#3 | 5'-GUACGAGACUCGGAACAAATT-3' |
|  | 5'-UUUGUUCCGAGUCUCGUACTT-3' |

**Supplementary Table. 2** Primers for real-time PCR

| Gene | Primer Sequences |
| --- | --- |
| CASP4 | Forward: 5'-TCCGAATATGGAGGCTGGAC-3' |
|  | Reverse:5'-CGTGTGCGGTTGTTTCTC-3' |
| NLRP1 | Forward: 5'-AACTCTTGTGCGTGCCTTCT-3' |
|  | Reverse:5'- TCACAAAGCAGAGACCCGTG-3' |
| FASN | Forward: 5'-AAACGGCAACCTGGTAGTGAG-3' |
|  | Reverse: 5'-GTGTCCATGAAGCTCACCCA-3' |
| ACC | Forward: 5'-GCTGCTCGGATCACTAGTGAA-3' |
|  | Reverse: 5'-TTCTGCTATCAGTCTGTCCAG-3' |
| SREBP-1 | Forward: 5'-GATGCGGAGAAGCTGCCTAT-3' |
|  | Reverse: 5'-GCTGTGTTGCAGAAAGCGAA-3' |
| SREBP-2 | Forward: 5'-CGGGCGCAACGCAAACAT-3' |
|  | Reverse: 5'-GTGACCTGGGTGAATGACCG-3' |
| GAPDH | Forward: 5'-GGTGAAGGTCGGAGTCAACG-3' |
|  | Reverse: 5'-TGGGTGGAATCATATTGGAACA-3' |

**Supplementary Figure.**
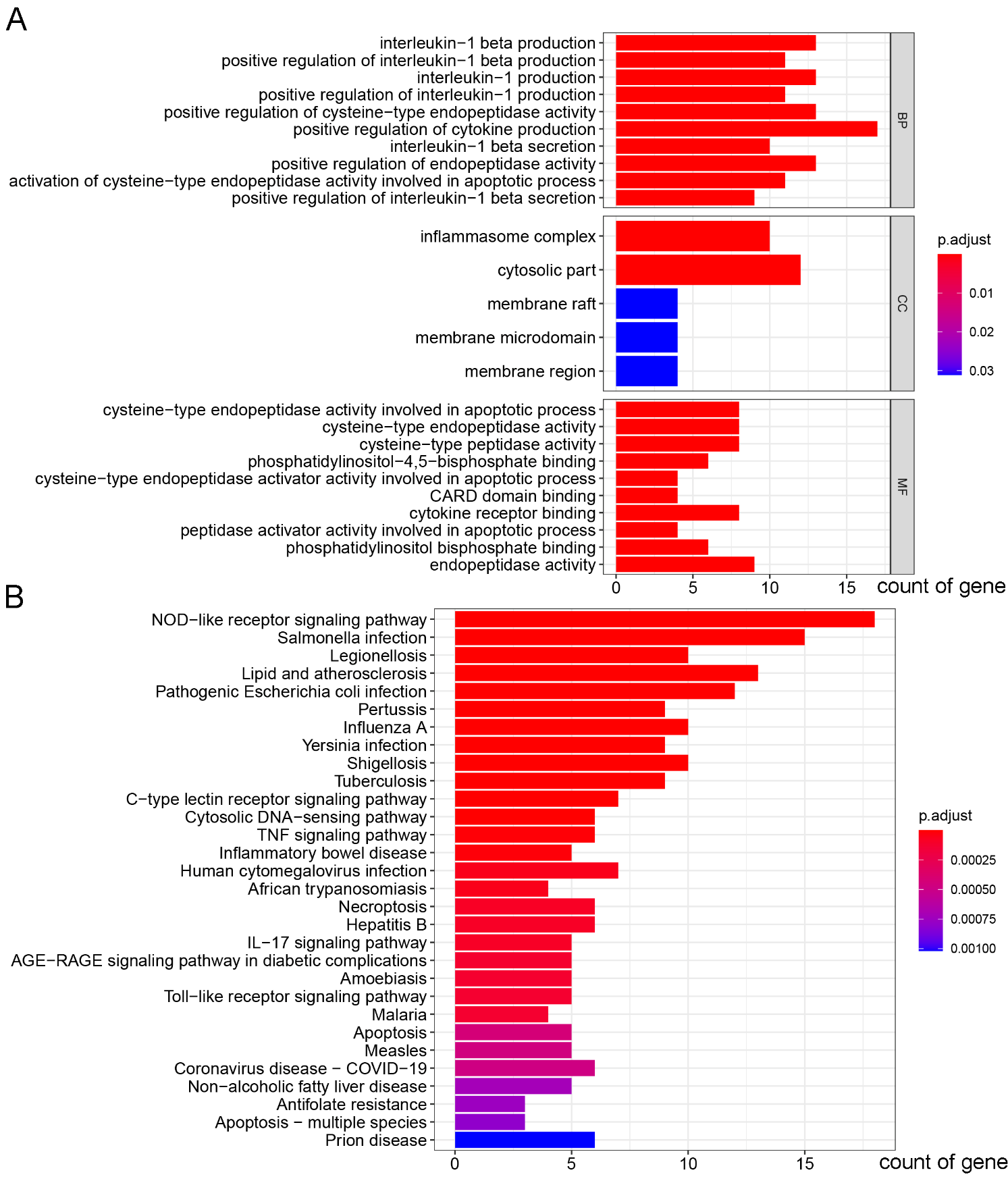
 **1** GO and KEGG pathway enrichment analysis of 319 glycolysis-related genes selected from GSEA (A) The bar plot of GO pathway enrichment analysis. (B) The bar plot of KEGG pathway enrichment analysis. (BP: biological process; CC: cell component; MF: molecular function)

**Supplementary Figure.**
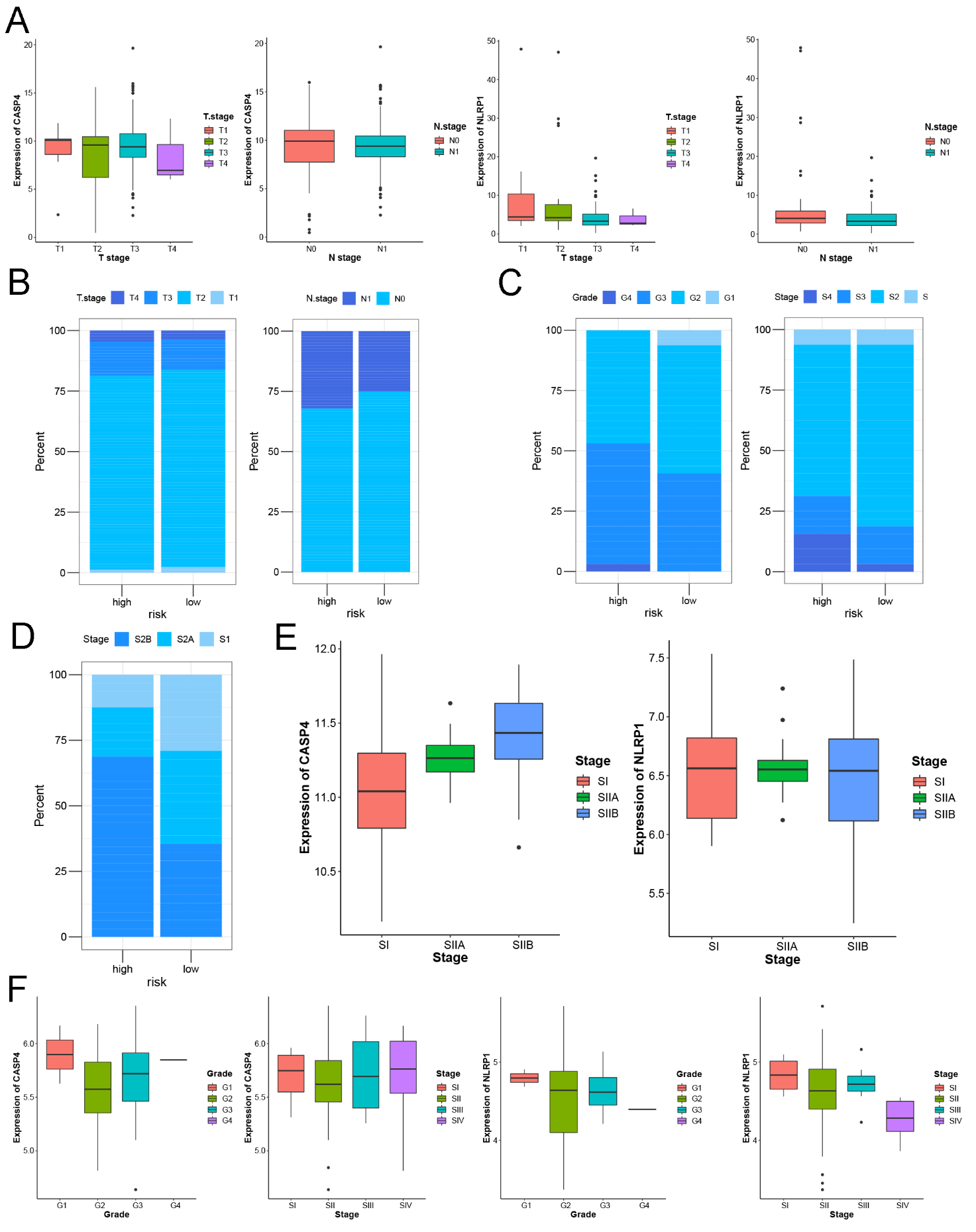
**2** The effects of the genes and the risk score on the clinical presentation of the patients (A) Differences in the expression levels of CASP4 and NLRP1 in tumors with different T and N stages in TCGA-PAAD cohort. (B-D) Clinical presentation distribution of tumors at different risk in TCGA-PAAD, GSE57495 and GSE62452 cohorts. (E-F) Differences in the expression levels of CASP4 and NLRP1 in tumors with different stage and grade in GSE57495 and GSE62452 cohorts.

**Supplementary Figure. 3** NLRP1 expression level did not affect the number of lipid droplets in PANC-1 and Aspc-1 cells. The mean number of lipid vesicles per cell. The lipid droplets were counted randomly (≥50 cells were counted per condition). (*P<0.05, **P<0.01, ***P<0.001)
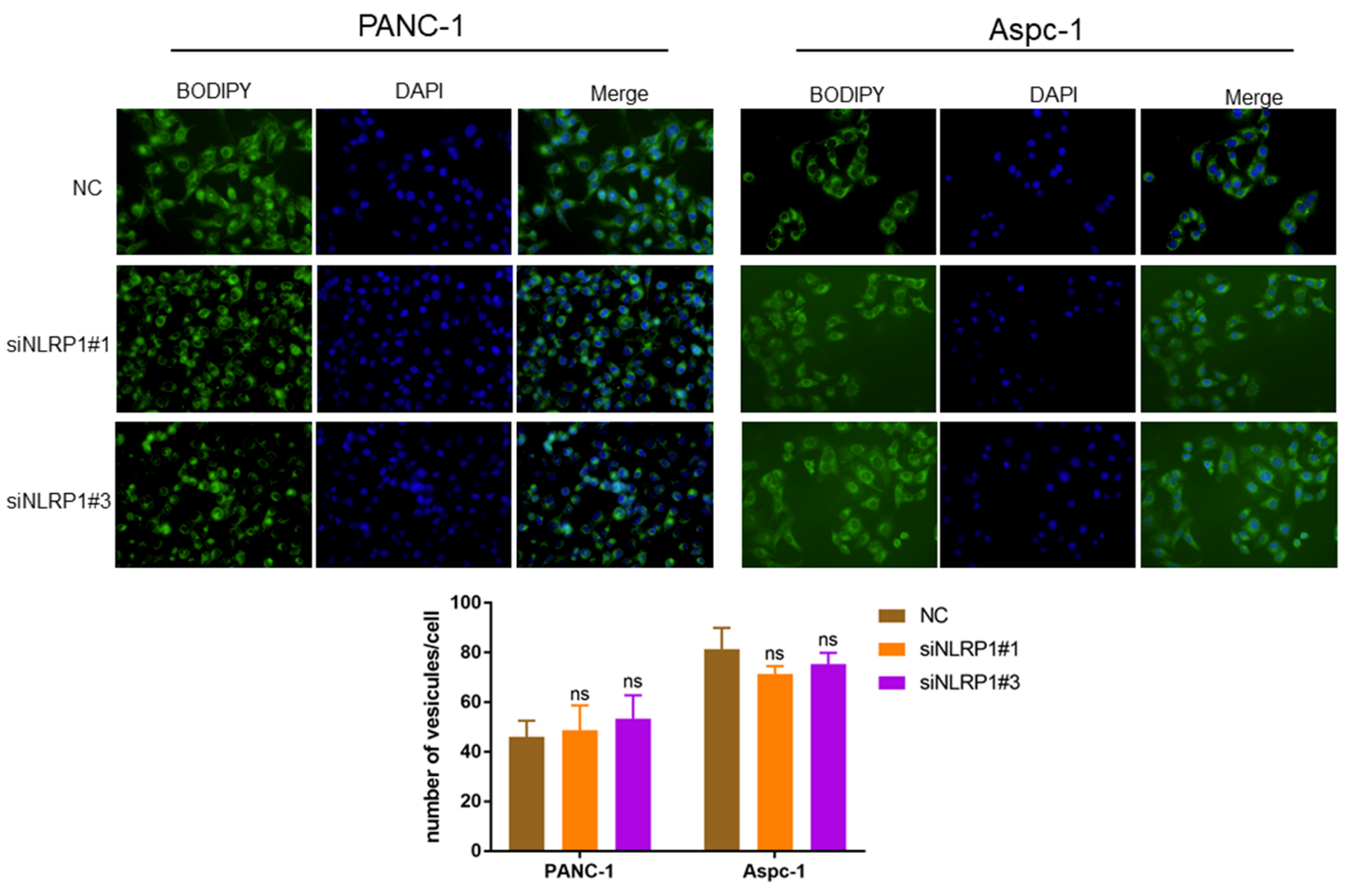

Supplement: Supplementary file 1 — (DOCX 1933.8 kb) [file 12672_2022_495_MOESM1_ESM.docx]
